# Naturally Occurring Isoleucyl-tRNA Synthetase without tRNA-dependent Pre-transfer Editing*

**DOI:** 10.1074/jbc.M115.698225

**Published:** 2016-02-26

**Authors:** Nevena Cvetesic, Morana Dulic, Mirna Bilus, Nikolina Sostaric, Boris Lenhard, Ita Gruic-Sovulj

**Affiliations:** From the ‡ Department of Chemistry, Faculty of Science, University of Zagreb, Horvatovac 102a, 10000 Zagreb, Croatia and; the § Computational Regulatory Genomics Group, MRC Clinical Sciences Centre, Imperial College London, London W12 0NN, United Kingdom

**Keywords:** aminoacyl tRNA synthetase, antibiotic resistance, protein evolution, protein synthesis, transfer RNA (tRNA), isoleucyl-tRNA synthetase, mupirocin, proofreading, tRNA-dependent pre-transfer editing

## Abstract

Isoleucyl-tRNA synthetase (IleRS) is unusual among aminoacyl-tRNA synthetases in having a tRNA-dependent pre-transfer editing activity. Alongside the typical bacterial IleRS (such as *Escherichia coli* IleRS), some bacteria also have the enzymes (eukaryote-like) that cluster with eukaryotic IleRSs and exhibit low sensitivity to the antibiotic mupirocin. Our phylogenetic analysis suggests that the *ileS1* and *ileS2* genes of contemporary bacteria are the descendants of genes that might have arisen by an ancient duplication event before the separation of bacteria and archaea. We present the analysis of evolutionary constraints of the synthetic and editing reactions in eukaryotic/eukaryote-like IleRSs, which share a common origin but diverged through adaptation to different cell environments. The enzyme from the yeast cytosol exhibits tRNA-dependent pre-transfer editing analogous to *E. coli* IleRS. This argues for the presence of this proofreading in the common ancestor of both IleRS types and an ancient origin of the synthetic site-based quality control step. Yet surprisingly, the eukaryote-like enzyme from *Streptomyces griseus* IleRS lacks this capacity; at the same time, its synthetic site displays the 10^3^-fold drop in sensitivity to antibiotic mupirocin relative to the yeast enzyme. The discovery that pre-transfer editing is optional in IleRSs lends support to the notion that the conserved post-transfer editing domain is the main checkpoint in these enzymes. We substantiated this by showing that under error-prone conditions *S. griseus* IleRS is able to rescue the growth of an *E. coli* lacking functional IleRS, providing the first evidence that tRNA-dependent pre-transfer editing in IleRS is not essential for cell viability.

## Introduction

Aminoacyl-tRNA synthetases (aaRS)^3^ establish the genetic code through the specific attachment of amino acid to their cognate tRNA. Because of their essential role in translation of the genetic information, these enzymes are thought to be among the most ancient of extant proteins. Aminoacylation proceeds in two steps, both catalyzed by the same synthetic site of the corresponding aaRS. An amino acid is activated to aminoacyl-adenylate intermediate (aa-AMP) at the expense of ATP, followed by transfer of aminoacyl moiety to the 2′- or 3′-OH groups of the terminal adenosine of the cognate tRNA.

**FIGURE 1.**
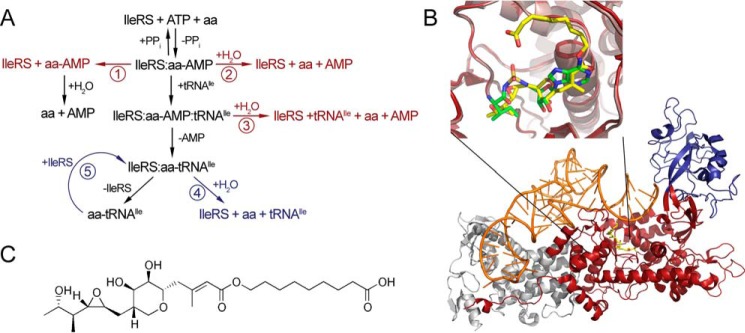
**Structure of enzymatic pathway of IleRS.** *A,* schematic presentation of enzymatic reactions. The central pathway, colored in *black*, represents amino acid activation, tRNA binding, aminoacyl transfer, and dissociation of aminoacylated tRNA from the enzyme. Editing pathways are shown to the *left* and *right*. Pre-transfer editing may proceed through enhanced dissociation of non-cognate aminoacyl-AMP (*pathway 1*) or through its enzymatic hydrolysis, which may be tRNA-independent (*pathway 2*) or tRNA-dependent (*pathway 3*). Misaminoacylated tRNA is deacylated through post-transfer editing, in *cis* (*pathway 4*) or in *trans* (*pathway 5*). Editing reactions occurring in synthetic site are colored in *red*, and the ones taking place in the editing domain are colored in *blue. B,* structure of *S. aureus* IleRS in complex with mupirocin and tRNA^Ile^ (Protein Data Bank code 1FFY). Synthetic domain is colored in *red*, editing domain in *blue*, tRNA^Ile^ in *orange*, and mupirocin in *yellow*. The *inset* shows overlapped structures *T. thermophilus* synthetic site with mupirocin in *yellow* (Protein Data Bank code 1JZS) and Ile-AMS in green (Protein Data Bank code 1JZQ). *C,* mupirocin structure.

The fidelity of aminoacyl-tRNA (aa-tRNA) synthesis is prerequisite for accurate transmission of the genetic message into protein assemblies. For many aaRSs, the main challenge in coupling of cognate amino acid-tRNA pairs is to discriminate against structurally and chemically similar non-cognate amino acids. When this task cannot be accomplished in the synthetic reaction alone, aaRSs take advantage of error-correction mechanisms (1, 2). Using a complex network of editing pathways (Fig. 1*A*), these enzymes proofread both the reaction intermediate (aa-AMP) and the final product, aminoacylated tRNA. The synthetic site provides the first correction point, catalyzing hydrolysis of the non-cognate aa-AMP (pre-transfer editing) (3, 4). A considerable number of aaRSs exhibit weak pre-transfer editing of ambiguous relevance in the absence of tRNA (Fig. 1*A, pathway 2*). Pre-transfer editing may be further stimulated by tRNA (Fig. 1*A, pathway 3*). This pathway is seldom used by aaRSs and is clearly demonstrated thus far only in isoleucyl-tRNA synthetase (IleRS) from *Escherichia coli* (see below). Finally, the selective release pathway may eliminate weakly bound non-cognate aa-AMPs prone to dissociation (Fig. 1*A, pathway 1*). Frequently, the non-cognate amino acid evades pre-transfer editing and gets transferred to tRNA. A misaminoacylated tRNA is then rapidly hydrolyzed in a separate well defined aaRS domain dedicated for this activity. In post-transfer editing (Fig. 1*A, pathway 4*), translocation of the aminoacylated 3′-end of the tRNA from the synthetic to the distant editing site positions the amino acid for proofreading. This pathway dominates in editing aaRSs because dedication of the editing site for post-transfer editing enabled its evolution toward high efficiency and specificity.

IleRS is responsible for decoding of isoleucine codons in all three domains of life (5). Besides isoleucine, IleRS also activates non-cognate valine with a discrimination factor as low as 200, and thus it requires editing to enhance accuracy of isoleucyl-tRNA^Ile^ (Ile-tRNA^Ile^) synthesis (6, 7). IleRS is a class I aaRS enzyme built around the conserved N-terminal Rossmann fold catalytic domain, which encloses the synthetic site (8). A peptide termed connective peptide 1 (CP1) is inserted between the two halves of the catalytic domain and folds into an independent domain that hosts the post-transfer editing site (8–10). This feature is shared with closely related class I valyl- (11) and leucyl-tRNA synthetases (ValRS and LeuRS, respectively) (12–14). IleRS from *E. coli* (EcIleRS) is characteristic for its tRNA-dependent pre-transfer editing and thus has served as a model system to address the mechanism of this idiosyncratic proofreading pathway (3, 4, 7, 15). We recently demonstrated that this pathway represents a minor pathway, contributing up to 30% of total editing (4).

IleRS of many bacteria and all eukaryote mitochondria share characteristic sequence elements, including the C-terminal peptide containing a conserved zinc-binding motif essential for aminoacylation (16). The second IleRS type, found in the eukaryotic cytoplasm and also in a number of bacterial species (so-called eukaryote-like IleRS), lacks the Cys-4 cluster at the C terminus and has unique sequence elements throughout the primary structure. Eukaryotic and eukaryote-like enzymes are less susceptible to inhibition by the natural product pseudomonic acid A, the dominant component of the antibiotic mupirocin-produced by *Pseudomonas fluorescens* (17). The finding that some bacteria have IleRSs that closely resemble the enzymes from eukaryotes was taken as an indication for the horizontal gene transfer from eukarya to bacteria (18–21).

Mupirocin manifests its antimicrobial activity by high affinity binding to the synthetic site of only bacterial IleRS (Fig. 1, *B* and *C*). The tyrosine residue in the synthetic site of the bacterial enzymes that is critical for tRNA-dependent pre-transfer editing in EcIleRS (Tyr-59 (4)) is replaced with phenylalanine or threonine in eukaryotic/eukaryote-like IleRSs. With these considerations in mind, we sought to explore whether the synthetic sites of the evolutionarily distinct IleRS types have diverged in catalytic features and antibiotic resistance. Cytosolic IleRS from yeast *Saccharomyces cerevisiae* (ScIleRS) and the eukaryote-like enzyme from *Streptomyces griseus* (SgIleRS) were used as model enzymes of the eukaryotic/eukaryote-like type. We show that SgIleRS surprisingly lacks tRNA-dependent pre-transfer editing, although the yeast enzyme exploits that pathway to a considerable extent. This work provides the first evidence that tRNA-dependent pre-transfer editing is not an evolutionary constraint for the IleRS synthetic site and shows that the accuracy of Ile-tRNA^Ile^ synthesis may be entirely ensured by the powerful post-transfer editing domain, which is absolutely conserved through evolution.

## Experimental Procedures

### 

#### 

##### Production and Purification of IleRS Enzymes

*ileS* amplification from *S. griseus* genomic DNA was performed using a modified touchdown PCR in the presence of 5% DMSO due to the high GC content of the *S. griseus* genome. Wild type (WT) and D334A SgIleRS enzymes were expressed from pET28b in *E. coli* BL21 (DE3) cells. The culture was grown at 30 °C until mid-exponential phase was reached, and expression was induced overnight at 15 °C. During growth (at *A*_600_ 0.1–0.2), ZnCl_2_ was added to a final concentration of 1 mm to ensure formation of the proper zinc-bound conformation. Each enzyme was purified by standard Ni^2+^-NTA chromatography, and their purities were estimated on SDS gels as greater than 98%.

WT and D333A ScIleRS were expressed from pET28b similarly as described for SgIleRS. The difference was in the use of *E. coli* Rosetta (DE3) cells and induction of IleRS expression overnight at 18 °C. Purification was performed using Ni^2+^-NTA resin, followed by size-exclusion chromatography to separate the active monomers from the inactive aggregates. The mobile phase consisted of 50 mm HEPES-KOH, pH 7.5, 500 mm NaCl, 5 mm β-mercaptoethanol, and 5% glycerol. Monomeric fractions were pooled and concentrated to 6–8 mg/ml, and glycerol was added up to 10% prior storage at −80 °C. Under these conditions, the monomeric form was stable and did not reform aggregates.

Small amounts of ScIleRS were produced in *S. cerevisiae* strain S2088α by overexpression upon galactose induction from pCJ11 and purified by standard Ni^2+^-NTA chromatography. Kinetic properties of the ScIleRS produced in *E. coli* were comparable with the ones of ScIleRS produced in *S. cerevisiae*, as tested in ATP-PP_i_ exchange assay, aminoacylation, and editing in the presence of tRNA.

##### Purification of tRNA^Ile^ from S. griseus and S. cerevisiae

*S. griseus* or *S. cerevisiae* cells were grown at 30 °C until saturation in the modified YEME media or YPD medium, respectively, and total tRNA was isolated using a standard phenol/chloroform procedure. A particular tRNA^Ile^ isoacceptor was then isolated from the total tRNA using a selective hybridization method (22). The 3′-biotinylated oligo-DNA probes complementary to the *S. griseus* tRNA_GAU_^Ile^ (SgtRNA^Ile^) or *S. cerevisiae* tRNA_IAU_^Ile^ (SctRNA^Ile^) major isoacceptor sequences were designed to include the anticodon loop and D-arm (SgtRNA^Ile^ oligonucleotide, ATCAGGGATGCGCTCTAACCAACTGAG, and SctRNA^Ile^ oligonucleotide, ATTAGCACGGTGCCTTAACCAACTGGG).

Unfractionated SgtRNA was mixed with the resin (the probes bound to streptavidin-Sepharose) in the standard hybridization buffer (final concentrations 10 mm Tris-Cl, pH 7.5, 0.9 m NaCl, and 0.1 mm EDTA); tRNA was heat-denatured for 10 min at 65 °C, and the suspension was allowed to slowly cool to ambient temperature to enable tRNA-DNA oligo hybridization. The resin was washed to remove the unbound tRNA, and the bound tRNA molecules were eluted in 10 mm Tris, pH 7.5 by heating to 65 °C. The eluted SgtRNA^Ile^ exhibited ∼85% acceptor activity.

Unfractionated SctRNA^Ile^ and the resin were mixed in the modified hybridization buffer that contained 10 mm Tris-Cl, pH 7.5, 0.9 m tetraethylammonium chloride, and 0.1 mm EDTA. The eluted fractions exhibited ∼35% isoleucine acceptor activity. SctRNA^Ile^ was further purified using reverse phase chromatography (Jupiter C4 column, 4.6 × 250 mm, Phenomenex). The sample enriched with SctRNA^Ile^ (35% acceptor activity) was isoleucylated using WT ScIleRS. Aminoacylated tRNA was extracted using acidic phenol, ethanol-precipitated, and dialyzed against 5 mm NaOAc, pH 4.5. The attachment of isoleucine to SctRNA^Ile^ increases its retention time and provides the basis for separation from other tRNA species. The column was equilibrated with buffer A (20 mm NH_4_OAc, pH 5.0, 10 mm Mg(OAc)_2_, 400 mm NaCl), and the retained molecules were eluted with the programmed linear gradients of buffer B (buffer A with 30% v/v ethanol). Fractions containing aminoacylated tRNAs were pooled, deacylated in Tris-Cl, pH 8.8, ethanol-precipitated, and dialyzed against 10 mm HEPES-KOH, pH 7.5. Final SctRNA^Ile^ sample exhibited 90% acceptor activity.

##### Preparation of Radiolabeled tRNA

[^32^P]tRNA^Ile^ was prepared as described (23). Aminoacylated or misaminoacylated [^32^P]tRNA^Ile^ was prepared by mixing 10 μm [^32^P]SgtRNA^Ile^ or [^32^P]SctRNA^Ile^ with 3 μm D334A SgIleRS or D333A ScIleRS, 4 mm ATP, 0.004 units/μl IPPase, and 4 mm valine or isoleucine in 75 mm HEPES-KOH, pH 7.5, 20 mm MgCl_2_, 5 mm DTT. The aa-[^32^P]tRNA^Ile^ was extracted as described previously (14, 24) and renatured prior to use by heating to 80 °C, adding MgCl_2_ to a 10 mm final concentration, and slow cooling to ambient temperature.

##### Standard Kinetic Methodology

ATP-PP_i_ exchange and reactions where [^32^P]tRNA^Ile^ or aa-[^32^P]tRNA^Ile^ were followed were quenched using a mixture of NaOAc (pH 4.5, 0.5 m final concentration) and SDS (final 0.1%). In ATP-PP_i_ exchange assay, radiolabeled ATP and PP_i_ were separated on TLC plates using 750 mm KH_2_PO_4_, pH 3.5, and 4 m urea as a mobile phase. Fraction of formed radioactive ATP was monitored in time to determine activation rate constant. In aminoacylation and deacylation assays, the fraction of aa-[^32^P]tRNA^Ile^ was followed in time. Quenched reaction aliquots were directly degraded using P1 nuclease (final concentration 0.013 units/μl, 1 h at room temperature), and aa-[^32^P]tRNA^Ile^ and [^32^P]tRNA^Ile^ degradation products were separated by TLC in 0.1 m ammonium acetate and 5% acetic acid, as described previously (3). Time points taken from the kinetic reactions that followed [^32^P]AMP formation were quenched in formic acid (1 m final concentration) and analyzed by TLC, as described for aminoacylation assays (3, 25). All quantitation was performed by phosphorimaging, and the data were analyzed using GraphPad Prism.

##### Kinetic Measurements in Single Turnover

Single-turnover reactions were measured using the KinTek RQF-3 instrument or by hand sampling at 30 °C. Reactions were quenched with NaOAc (pH 4.5, final concentration 0.8 m), and the collection tubes contained SDS (final w/v 0.1%). To measure the transfer step, the IleRS·aa-AMP complexes were preformed *in situ* by incubation of 20–30 μm D334A SgIleRS or D333A ScIleRS with 5 mm isoleucine or 16 mm valine in the standard IleRS reaction buffer supplemented with 8 mm ATP and 0.008 units/μl IPPase for 5 min at 30 °C, and the reaction was started by mixing with [^32^P]tRNA^Ile^. The data were fit to the single exponential equation, *y* = *Y*_0_ + *A* × (1 − e^−*k*trans ×^ *^t^*), where *Y*_0_ is the *y* intercept; *A* is the amplitude; *k*_trans_ is the apparent transfer rate constant, and *t* is time. Single-turnover deacylation steps were measured by mixing equal volumes of 100–500 nm valyl- or isoleucyl-[^32^P]tRNA^Ile^ with 20–30 μm IleRS in 125 mm HEPES-KOH, pH 7.5, 20 mm MgCl_2_, 5 mm DTT. The data were fit to the single exponential equation, *y* = *Y*_0_ + *A* × e^−*k*deacyl ×^ *^t^*, where *Y*_0_ is the *y* intercept; *A* is the amplitude; *k*_deacyl_ is the apparent deacylation rate constant, and *t* is time.

##### Activation of Amino Acids

ATP-PP_i_ exchange was measured at 30 °C in the reaction mixture containing 50 mm HEPES-KOH, pH 7.5, 20 mm MgCl_2_, 0.1 μg/μl BSA, 5 mm DTT, 4 mm ATP, and 1 mm [^32^P]PP_i_ (0.2–0.4 Ci/mol). The reactions were started by addition of amino acid. The enzymes were present at the concentration of 100 nm, and the concentration of amino acids was varied between 0.1 × *K_m_* and 10 × *K_m_*. Kinetic parameters were determined by fitting the data directly to the Michaelis-Menten equation.

##### Aminoacylation Assay

Aminoacylation reactions were performed in the reaction mixture containing 20 mm HEPES-KOH, pH 7.5, 100 μm EDTA, 150 mm NH_4_Cl, 10 μg/ml BSA, 10 mm MgCl_2_, 2 mm ATP, and 1 mm isoleucine at 37 °C in the case of EcIleRS or at 30 °C in the case of SgIleRS and ScIleRS. EctRNA^Ile^ and SgtRNA^Ile^ were present at a concentration of 15 μm (of which ∼20 nm was ^32^P-labeled tRNA^Ile^), and SctRNA^Ile^ was present at concentration of 9 μm in the mixture of total yeast tRNA. The reactions were started by addition of amino acid. The enzymes were present at the following concentrations: 20 nm EcIleRS with EctRNA^Ile^; 20 nm SgIleRS with EctRNA^Ile^; 50 nm ScIleRS with EctRNA^Ile^; 200 nm EcIleRS with SgtRNA^Ile^; 50 nm ScIleRS with SgtRNA^Ile^; 20 nm SgIleRS with SgtRNA^Ile^; 1 μm EcIleRS with SctRNA^Ile^; 50 nm ScIleRS with SctRNA^Ile^, and 250 nm SgIleRS with SctRNA^Ile^.

##### Parallel Formation of AMP and aa-tRNA^Ile^

The reactions were conducted at 30 °C in a buffer containing 50 mm HEPES-KOH, pH 7.5, 20 mm MgCl_2_, 5 mm DTT, 0.1 μg/μl BSA, 0.004 units/μl IPPase, and 200 μm ATP. The reaction mixture further contained 15 μm SgtRNA^Ile^ or 10 μm SctRNA^Ile^ and either 2 mm isoleucine or 20 mm valine. WT or D334A SgIleRS was present at 15 nm in the reactions with Ile and 50 nm in the reactions with Val. WT ScIleRS was 50 nm or 0.15 μm in the reactions with Ile or Val, respectively. D333A ScIleRS was 0.15 μm in reactions with both Ile and Val. Measurements were started by addition of amino acid. The reactions with SgIleRS additionally contained 40 μm EF-Tu activated as described previously (26), to prevent accumulation at the low plateau level of aa-tRNA^Ile^. The rates of aa-tRNA^Ile^ formation were the same with and without EF-Tu.

##### Complementation

Complementation was done on the LB or M9 plates containing 30 μg/ml kanamycin, 34 μg/ml chloramphenicol, 50 μm isopropyl 1-thio-β-d-galactopyranoside in the presence or absence of 100 μm mupirocin. M9 plates were used with no amino acids added to the media or were supplemented with 150 μm isoleucine, 150 μm leucine and 1, 4, or 10 mm valine. Isoleucine and leucine were included to alleviate the effect of high valine concentration on amino acid synthesis. The constructs electroporated in the Rosetta cells were as follows: empty pET28, pET28 EcIleRS, pET28 ScIleRS, and pET28 SgIleRS. The plates were grown for 24 h at 30 °C. Under these conditions, all three proteins were expressed as confirmed by SDS-PAGE analysis of the protein extracts of liquid cultures grown under exactly the same conditions (data not shown). Semi-quantitative complementation drop-test was performed by placing 100-μl drops of decimal dilutions in PBS buffer (−1 to −6) for each strain on LB or M9 plates. The surface of the plates was dried 5–6 min under laminar air flow, and the plates were incubated at 30 °C for 36 h.

##### Inhibition by Mupirocin

Mupirocin inhibition was studied in ATP-PP_i_ exchange reaction. To determine the *K_i_* values with respect to isoleucine, mupirocin was used at the following concentrations: 0, 20, 50, 100, and 150 μm for inhibition of ScIleRS and 0, 10, 20, 30, and 40 mm for inhibition of SgIleRS. Isoleucine concentration spans 0.25× *K_m_* to 8× *K_m_* range (2, 5, 10, 20, and 50 μm for ScIleRS or 3.9, 7.8, 15.6, 39, and 78 μm for SgIleRS). The ScIleRS was present at a concentration of 0.15 μm and SgIleRS at 0.1 μm. Inhibition constants were determined by fitting the data to competitive inhibition model by Global Fit using GraphPad Prism.

##### Phylogenetic Analysis

Eukaryote-like IleRS sequences were searched in Public databases using BLASTP with *Staphylococcus aureus* bIleRS2 and *S. griseus* as query sequences. Archaeal, eukaryotic, and bacterial sequences were chosen to present a uniform distribution of species in the taxonomic tree. Sequences from UniProtKB/Swiss-Prot or RefSeq database were preferred, if available. The final dataset contained 334 sequences. Multiple sequence alignment (MSA) was performed using the Muscle tool (27) via the EBI web server with standard parameters. The MSA was trimmed to remove the poorly aligned regions with trimAI program (28) via the Phylemon2 web server using the automated 1 method. The MSA contained 2497 columns prior to trimming and 936 columns after trimming. The phylogenetic trees were constructed using a maximum-likelihood method RAxML (29) via the web server CIPRES science gateway with JTT defined as the substitution model and GAMMA model of rate heterogeneity. Phylogenetic tree topologies were visualized using FigTree version 1.4.2. The phylogenetic tree constructed using the complete MSA of IleRS sequences or the trimmed MSA showed no significant differences in tree topology.

Evolution of IleRS gene family was inferred using the Mowgli algorithm for reconciliation of the IleRS gene tree and the corresponding fully dated species tree (30). The species tree published by Ciccarelli *et al.* (31) was retrieved using the Interactive Tree Of Life (32). IleRS gene tree and the species tree were pruned using the R-package Ape (33) to contain only the mutual taxa as leaf nodes. Mowgli was used with standard parameters (costs defined as duplication (D) = 2, transfer (T) = 3, and loss (L) = 1) along with the Nearest Neighbor Interchange edit operations set to operate on edges of the gene tree with a bootstrap value lower than 80. Both the cost parameters and the threshold bootstrap values were varied to test the robustness of the events presented in the reconciliation tree (D = 2–4, T = 3–5, and Bootstrap threshold = 80–95). Expectedly, raising the cost of the horizontal gene transfer produced meaningless duplications that are evolutionarily highly unlikely, while raising the cost of duplications produced a high number of unlikely horizontal gene transfers. The reconciliation trees were visualized using the Sylvx open-source reconciliation viewer.

## Results

### 

#### 

##### Phylogenetic Analysis Groups SgIleRS with the Eukaryote-like Enzymes

**FIGURE 2.**
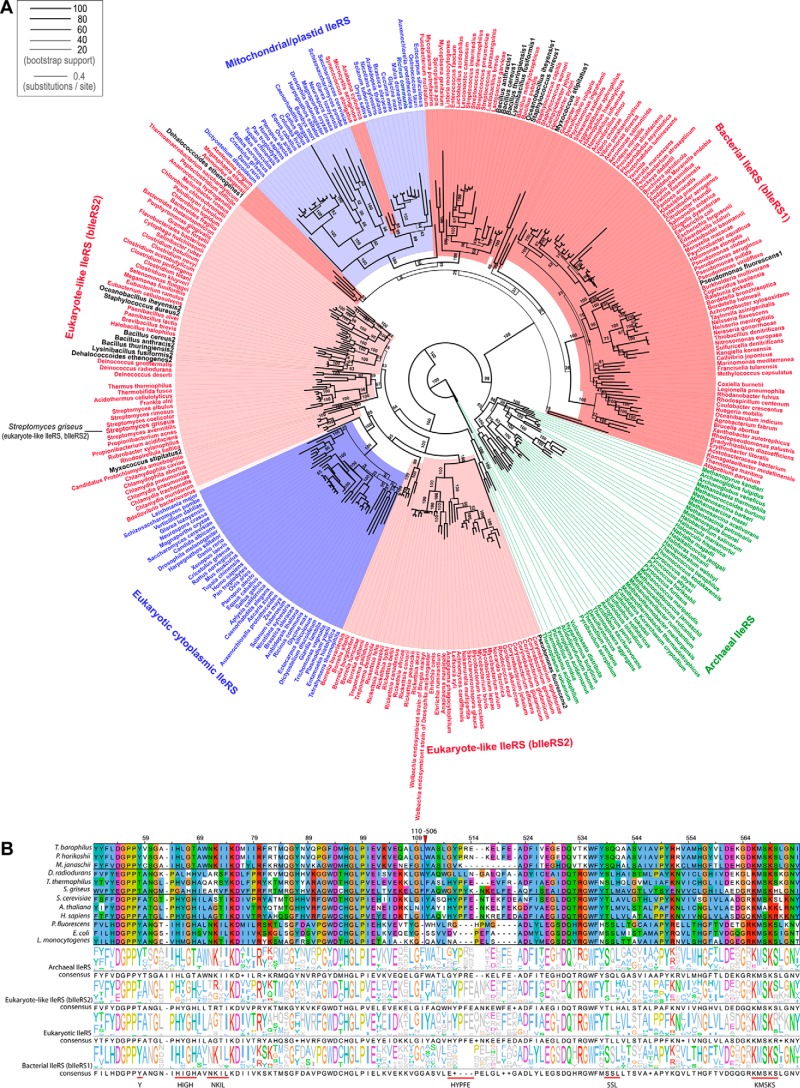
**IleRS phylogeny.** *A,* IleRS phylogenetic tree. Bacterial species are colored *red* (species with eukaryote-like IleRS are shown in *light red*); archaea are *green*, and eukaryotes are *blue* (mitochondrial/plastid IleRS are shown in *light blue*). Species with both bacterial IleRS (bIleRS1) and eukaryote-like IleRS (bIleRS2) are shown in *black*. The line width of each branch is scaled according to the bootstrap support value (see legend), and the *numbers* along the branches show the percentage reproducibility of nodes in bootstrap replicates (some values were omitted for simplicity). The *scale bar* represents 0.4 estimated amino acid substitutions per site. *B,* partial amino acid sequence alignment of representative archaeal, eukaryote-like (bIleRS2), eukaryotic, and bacterial (bIleRS1) IleRSs. The presented regions include the consensus sequences HIGH and KMSKS and characteristic bacterial (Tyr-59; ^71^NKIL^74^; ^572^SSL, numbering according to EcIleRS) or eukaryotic/eukaryote-like motifs (^541^HYPFE, numbering according to ScIleRS). Each sequence logo was determined using the complete alignment with 334 sequences and represents the conservation within a certain IleRS group. The sequences were assigned to a group according to the phylogenetic analysis. The *red triangle* marks the omitted portion of the alignment.

Actinomycetes and pseudomonads represent two of the major groups of bacteria found in soils and rhizospheres (34). Considering that the bacterium *S. griseus* may cohabit with mupirocin-producing organisms, SgIleRS is potentially interesting for investigating the impact of environmental pressure on enzyme synthetic site catalytic features. To determine the evolutionary history of SgIleRS, we extracted archaeal, eukaryotic, and bacterial IleRS sequences from public databases. The species were chosen to provide a uniform distribution across the taxonomic tree. A phylogenetic tree was constructed based on multiple alignments of the conserved portions of IleRSs from all domains of life, using the maximum likelihood-based phylogenetic inference method RAxML (29) (for details see “Experimental Procedures”). As expected, the analysis showed that the bacterial sequences group into two main clades (Fig. 2*A*) as follows: one containing the IleRS sequences that cluster with the eukaryotic enzymes and thus represent the so-called eukaryote-like lineage (herein named bIleRS2), and the second clade (herein named bIleRS1) clusters with mitochondrial IleRSs. The bIleRS2 type is found in Gram-positive bacteria, predominantly firmicutes and actinobacteria, but also in the chlamydiae, spirochaetes, and deinococcales. The prototypical bacterial enzyme (bIleRS1) is generally found in proteobacteria. The observed IleRS phylogeny is in good agreement with the results of previous analyses (20, 21, 35). We now have access to a substantially higher number of sequences, particularly of bacterial species. Accordingly, the eukaryote-like lineage is by this work cemented as a sizable clade that clusters more closely with their eukaryotic counterparts than with other bacterial enzymes.

The analysis clearly shows that the sequence of IleRS from the Gram-positive bacterium *S. griseus* belongs to the eukaryote-like enzymes and not to the typical bacterial IleRS clade (Fig. 2*A*). Inspection of the sequence alignment (Fig. 2*B*) shows differently conserved sequences of the catalytic domain among bacterial and eukaryote/eukaryote-like IleRSs. SgIleRS and other eukaryote-like enzymes together with IleRS of eukarya lack the sequence signatures of the following bacterial enzymes: Tyr-59; ^71^NKIL^74^; ^572^SSL (numbering according to EcIleRS). At the same time, the eukaryote/eukaryote-like group has its own distinguishing sequence motifs such as ^541^HYPFE (numbering according to ScIleRS).

Some members of the eukaryotic/eukaryote-like clade were previously characterized as less sensitive to mupirocin inhibition than bIleRS1 enzymes (36, 37). Here, we showed that SgIleRS shares this characteristic with other eukaryote-like (bIleRS2) enzymes (see below). Thus, both phylogenetic and biochemical analysis place SgIleRS within the bIleRS2 group.

##### Origin of Discrimination against Valine in the Synthetic Reaction Is Evolutionarily Conserved in IleRS

The *ileS2* gene follows significantly different phylogeny than *ileS1*, prompting us to investigate biochemical characteristics of the corresponding two IleRS types. The genes encoding SgIleRS and ScIleRS were cloned, and the proteins were overexpressed in *E. coli*. As a control, the yeast enzyme was also overexpressed in yeast. No significant difference in kinetic properties was observed between the enzymes produced in yeast or *E. coli*, thus permitting utilization of the heterologously expressed ScIleRS in this work (see under “Experimental Procedures”).

Amino acid activation in the presence of cognate isoleucine and non-cognate valine was followed using ATP-PP_i_ exchange assay to establish the proteins' misactivation frequencies expressed as discrimination factors ((*k*_cat_/*K_m_*_(cognate)_)/(*k*_cat_/*K_m_*_(non-cognate)_)). The discrimination factors of SgIleRS and ScIleRS were highly similar (118 and 150, Table 1). The observed low values manifest frequent misactivation, implicating a requirement for editing in eukaryotic and eukaryote-like IleRSs. ScIleRS and SgIleRS activated cognate and non-cognate amino acids with similar rates in a relatively fast step (Table 1). Both enzymes discriminated against valine solely at the ground state, with the binding step that contributes to the specificity about 100-fold. The results match our previous conclusions regarding discriminations against valine by EcIleRS (3).

**TABLE 1:** **Activation of isoleucine and valine by SgIleRS and ScIleRS at 30 °C** The values represent the best fit value ± S.E. of at least two independent experiments.

	*K_m_*/μmol dm^−3^	*k*_cat_/s^−1^	*k_cat_*/*K_m_*/s^−1^ μmol dm^−3^	Discrimination factor
SgIleRS + Ile	17.4 ± 3.0	20.6 ± 1.4	1.18	
SgIleRS + Val	1750 ± 130	19.3 ± 0.4	0.01	118
ScIleRS + Ile	7.6 ± 0.3	11.4 ± 0.8	1.5	
ScIleRS + Val	840 ± 10	8.6 ± 1	0.01	147

The second step of aminoacylation, transfer of aminoacyl moiety to tRNA, was investigated in both ScIleRS and SgIleRS. The enzyme was preincubated with a surplus of amino acid and ATP allowing formation of the aaRS·aminoacyl-AMP complex prior to mixing with a limiting amount of the corresponding [^32^P]tRNA in a rapid chemical quench instrument. Formation of aa-[^32^P]tRNA was followed in time, and the rate constant (*k*_trans_)_,_ which represents the chemical step of aminoacyl transfer or a slow conformational change that precedes the rapid chemistry step, was extracted (38). The data show that isoleucyl moiety is transferred to tRNA with the rates of 45 and 11 s^−1^ by wild-type yeast and *S. griseus* IleRS, respectively. The two-step aminoacylation followed under multiple-turnover conditions (Table 2) returned the rates significantly slower than the rates of the isolated amino acid activation or aminoacyl transfer step. This indicated that product release limits the rate of isoleucylation by the yeast and *S. griseus* enzyme.

**TABLE 2:** **Homologous and heterologous aminoacylation of ScIleRS, SgIleRS, and EcIleRS** The values represent the best fit value ± S.E. of at least two independent experiments.

	*k*_obs_/s^−1^
EcIleRS + EctRNA^Ile^	0.86 ± 0.2
EcIleRS + SgtRNA^Ile^	0.078 ± 0.002
EcIleRS + SctRNA^Ile^	0.0029 ± 0.0003
SgIleRS + EctRNA^Ile^	0.7 ± 0.2
SgIleRS + SgtRNA^Ile^	0.58 ± 0.03
SgIleRS + SctRNA^Ile^	0.061 ± 0.004
ScIleRS + EctRNA^Ile^	0.172 ± 0.003
ScIleRS + SgtRNA^Ile^	0.16 ± 0.03
ScIleRS + SctRNA^Ile^	0.68 ± 0.02

**FIGURE 3.**
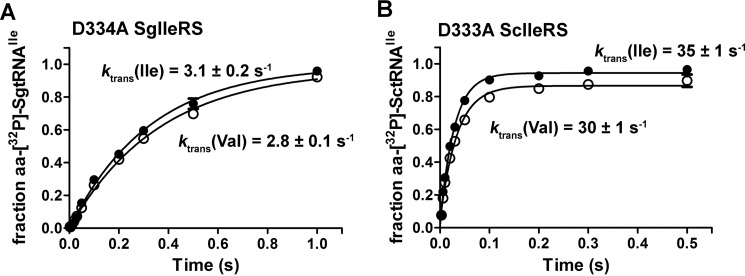
**Single-turnover transfer of isoleucine or valine by D334A SgIleRS (*A*) or D333A ScIleRS (*B*).** *Errors bars* correspond to the S.E. from three independent experiments.

To explore the specificity of aminoacyl transfer step, transfer of non-cognate valine to tRNA^Ile^ was monitored using the post-transfer editing-deficient IleRS to prevent valyl-tRNA^Ile^ (Val-tRNA^Ile^) hydrolysis. A highly conserved aspartate in the IleRS editing domain was previously recognized as the main determinant of post-transfer editing (15, 24). Hence, the D333A ScIleRS and D334A SgIleRS variants were produced, and their incapacity to deacylate Val-tRNA^Ile^ was independently confirmed (see below). Comparison of the *k*_trans_ obtained with isoleucine and valine using deacylation-defective enzymes (Fig. 3) demonstrated that neither of the IleRSs discriminated against valine at the second step of aminoacylation reaction. This parallels the previous findings on the *E. coli* enzyme (3) and is in a good agreement with the results on other editing aaRSs (14, 39). The D334A substitution influenced the rate of aminoacyl transfer step in SgIleRS (decreasing it from 11 to 3.1 s^−1^, see Fig. 3). The reason for such behavior is not clear yet. The effect imposed by analogous substitution has been previously observed for EcIleRS but only under multiple-turnover conditions (4). Taken together, our data show that the origin of discrimination against amino acids is well conserved in the synthetic reactions of both IleRS types.

##### Lack of tRNA-dependent Pre-transfer Editing in SgIleRS

The hydrolytic editing reactions regenerate amino acids and tRNAs, enabling their reuse in the next catalytic turnovers. In contrast, ATP is consumed in the aminoacylation/editing reactions generating AMP and PP_i_ (Fig. 1). Hence, non-stoichiometric accumulation of AMP above the level of aminoacylated tRNA is diagnostic of hydrolytic proofreading. Here, we took advantage of our recently developed approach (14) that monitors initial rates of [^32^P]AMP and aa-[^32^P]tRNA formation in parallel assays, one relying on [^32^P]ATP and another on [^32^P]tRNA, respectively. The extracted *k*_AMP_/*k*_Val-tRNA_ ratio reports ATP consumption per synthesized aa-tRNA. Furthermore, subtraction of the rate of aa-tRNA synthesis from the rate of AMP accumulation returns the rate of AMP formation related only to editing. This approach has proven highly valuable for the analysis of pre-transfer editing in EcIleRS (4).

Remarkably, we found that the post-transfer editing-defective variant of SgIleRS (D334A) displayed the similar rates of aminoacylation and AMP formation in the presence of valine, exhibiting a *k*_AMP_/*k*_Val-tRNA_ ratio of 1.1. Stoichiometric ATP consumption in Val-tRNA^Ile^ synthesis demonstrated the lack of proofreading by D334A SgIleRS, arguing against hydrolysis of Val-AMP alongside aminoacylation within the synthetic site. It thus appears that SgIleRS naturally lacks tRNA-dependent pre-transfer editing; a finding that implies that this pathway is optional in IleRS enzymes.

An equivalent analysis of the yeast enzyme showed a different outcome. Using post-transfer editing-defective D333A ScIleRS for parallel monitoring of AMP and Val-tRNA^Ile^ formation, the ratio of extracted rate constants was shown to be higher than 1 (*k*_AMP_/*k*_Val-tRNA_ = 2.19; Table 3), providing evidence of the tRNA-dependent pre-transfer editing activity. Alongside AMP, tRNA-dependent accumulation of Val-AMP with the rate constant of 0.021 ± 0.004 s^−1^ was also noticed. Previously determined solution-based Val-AMP hydrolysis (0.002 s^−1^ (3)) is 25-fold slower than the rate of AMP formation assigned to editing in D333A ScIleRS (*k*_ed_ = *k*_AMP_ − *k*_AA-tRNA_ = 0.049 s^−1^; Table 3). Thus, non-enzymatic hydrolysis only weakly contributes to AMP accumulation. Accordingly, the main tRNA-dependent pre-transfer editing pathway in ScIleRS is the enzyme-based aa-AMP hydrolysis. The new finding is that in yeast, tRNA may stimulate Val-AMP dissociation from the enzyme. The wild-type enzymes from *S. griseus* and the yeast cytosol returned the ratio *k*_AMP_/*k*_Val-tRNA_ of ∼6 and 23, respectively, due to the active CP1 post-transfer editing domain (Table 3). In the absence of tRNA, both ScIleRS and SgIleRS displayed very weak editing (0.002 and 0.004 s^−1^, Table 3), which is 10-fold slower than in *E. coli* IleRS (4).

**TABLE 3:** **Parallel formation of AMP and aa-tRNA by SgIleRS and ScIleRS and their deacylation variants** The values represent the best fit value ± S.E. of three independent experiments.

	*k*_(AMP)_/s^−1^	*k*_(aa-tRNA)_/s^−1^	*k*_(AMP)_/*k*_(aa-tRNA)_	*k*_tRNA-ind_/s^−1^
SgIleRS WT + Ile*^a^*	0.64 ± 0.08	0.65 ± 0.05	0.98	ND*^b^*
SgIleRS WT + Val*^a^*	0.83 ± 0.2	0.13 ± 0.02	6.24	0.004
SgIleRS D334A + Ile*^a^*	0.20 ± 0.02	0.19 ± 0.02	1.05	ND
SgIleRS D334A + Val*^a^*	0.20 ± 0.04	0.19 ± 0.04	1.05	0.004
ScIleRS WT + Ile	0.20 ± 0.03	0.13 ± 0.02	1.60	ND
ScIleRS WT + Val	0.53 ± 0.08	0.023 ± 0.007	23	0.002
ScIleRS D333A + Ile	0.038 ± 0.006	0.030 ± 0.008	1.27	ND
ScIleRS D333A + Val	0.09 ± 0.01	0.041 ± 0.012	2.19*^c^*	0.002

*^a^* The reactions with SgIleRS were measured in the presence of EF-Tu. In the absence of EF-Tu, aa-tRNA accumulated at lower concentrations (down to 10% of the product), depending on the IleRS concentration added.

*^b^* ND means not determined due to insufficient activity.

*^c^* A portion of Val-AMP accumulates in solution with rate *k*(Val-AMP) = 0.021 ± 0.004 s^−1^ participating in the selective release pathway. If both Val-AMP and AMP are taken into account, the ratio of used ATP per aa-tRNA is raised to 2.6.

##### SgIleRS Synthetic Site Is Highly Resistant to Mupirocin

**FIGURE 4.**
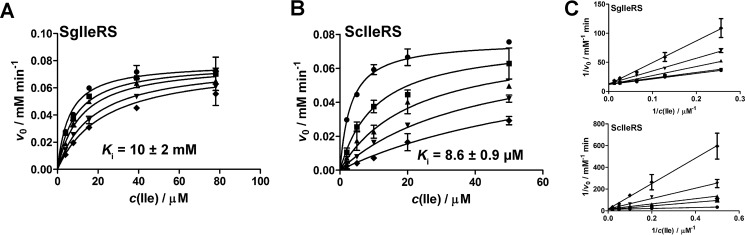
**Inhibition of isoleucine activation by mupirocin in the reactions by SgIleRS or ScIleRS.** *A,* activation by SgIleRS using 3.9, 7.8, 15.6, 39, and 78 μm isoleucine and 0 (●), 5 (■), 10 (▴), 20 (▾), and 30 mm (♦) mupirocin. *B,* activation by ScIleRS using 2 (●), 5 (■), 10 (▴), 20 (▾), and 50 μm (♦) isoleucine and 0, 20, 50, 100, and 200 μm mupirocin. The inhibition constants were determined by fitting the data to competitive inhibition model using GraphPad Prism. The values represent the best fit value ± S.E. of two independent experiments. *C,* Lineweaver-Burk plots depict a competitive inhibition pattern for both enzymes.

Finding that yeast IleRS exhibits tRNA-dependent pre-transfer editing indicates that this quality control step is preserved in the eukaryotic/eukaryote-like lineage (Fig. 2*A*). Hence, it is intriguing that SgIleRS is deprived of this activity. Given that *S. griseus* is a soil bacterium that may experience competition for the biological niche with mupirocin-producing *P. fluorescens*, we hypothesized that a need for superior antibiotic resistance has driven evolution of the SgIleRS synthetic site toward higher mupirocin resistance at the expense of its tRNA-dependent pre-transfer editing. Mupirocin is a strong competitive inhibitor of EcIleRS (bIleRS1) with respect to both isoleucine and ATP, which displays highly similar inhibition constants in amino acid activation and two-step overall aminoacylation (2–5 nm (40)). To address our hypothesis, we measured the antibiotic's inhibition constants in both the yeast and *S. griseus* enzymes. Steady-state kinetic analysis revealed that pyrophosphate exchange reaction in ScIleRS and SgIleRS is competitively inhibited by mupirocin, albeit with significantly different inhibition constants with respect to isoleucine. The yeast enzyme exhibited good resistance with the *K_i_* = 28 μm (*K_m_*_, Ile_ =6 μm; Fig. 4), which is in agreement with the *K_i_* value previously determined in the two-step aminoacylation reaction by the yeast enzyme (36). Remarkably, SgIleRS displayed a 300-fold higher inhibitory constant than the yeast enzyme with *K_i_* being as high as 10 mm (*K_m_*_, Ile_ = 4.8 μm; Fig. 4). Considering that the mupirocin-resistant IleRS from *P. fluorescens* displays *K_i_* in the same millimolar range (41), our data demonstrated that SgIleRS provides *S. griseus* a similar level of protection against mupirocin as reached in the mupirocin-producing *P. fluorescens*.

##### tRNA-dependent Pre-transfer Editing Is Not Essential for E. coli Viability

**FIGURE 5.**
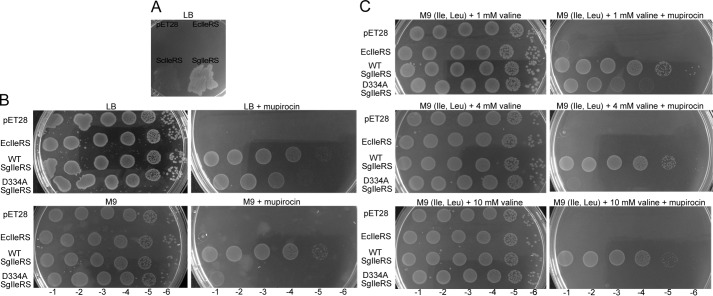
**Complementation of *E. coli* growth in the presence of 100 μm mupirocin by expression of ScIleRS or SgIleRS in various conditions.** In all experiments, the expression of ScIleRS, SgIleRS, or EcIleRS from pET28 plasmid was induced by 50 μm isopropyl 1-thio-β-d-galactopyranoside. *A,* complementation of *E. coli* growth on LB plates by expression of additional EcIleRS, ScIleRS, or SgIleRS. Semi-quantitative complementation drop-test of *E. coli* growth was performed by adding 100-μl decimal dilutions (−1 to −6) of each strain to the following plates. *B,* LB plates with or without mupirocin and M9 plates (no amino acids added) with or without mupirocin. *C,* M9 plates supplemented with 150 μm Ile, 150 μm Leu, and 1, 4, or 10 mm Val with or without mupirocin.

The loss of tRNA-dependent pre-transfer editing in SgIleRS specifies this pathway as dispensable, at least in *S. griseus*. To find out whether this is a more general phenomenon, we tested whether SgIleRS may complement IleRS function in *E. coli*. The prerequisite for complementation was met as SgIleRS aminoacylated well EctRNA^Ile^ (Table 2). Using mupirocin to inhibit EcIleRS and thus arrest protein synthesis and bacterial growth, we tested the ability of SgIleRS to functionally substitute the inhibited EcIleRS. Mupirocin, present at 100 μm in LB plates, completely inhibited growth of *E. coli* Rosetta strain (Novagen), but expression of SgIleRS effectively rescued the growth (Fig. 5*A*). To examine the cellular requirement for tRNA-dependent pre-transfer editing, we searched for error-prone conditions where editing is essential for *E. coli* growth. In doing so, the viability of *E. coli* cells transformed with the WT or editing-deficient SgIleRS (D334A) was followed using a semi-quantitative drop test in the presence of mupirocin (see “Experimental Procedures”). Both strains exhibited highly similar viability on LB plates with or without mupirocin, indicating that these conditions do not promote mistranslation (Fig. 5*B*). Next, we tested the viability on minimal plates to show that, under limiting amino acid conditions, only the WT SgIleRS complemented *E. coli* growth. To promote the controlled error-prone conditions, we supplemented M9 plates with the various concentrations of valine (Fig. 5*C*). We found that expression of editing-deficient SgIleRS was able to support the growth only in the presence of 1 mm valine. At the higher concentrations of valine (4 or 10 mm), the growth was rescued exclusively by expression of WT SgIleRS. Because this enzyme is deprived of tRNA-dependent pre-transfer editing, our results provide evidence that this proofreading step is not important under editing-required conditions. Moreover, the strains exhibited highly similar viability on minimal plates with or without the addition of 4 mm valine, 150 μm leucine, and 150 μm isoleucine. This result substantiates the physiological relevance of the complementation experiment as the amino acid supplement was chosen to mimic the experimentally determined natural intracellular amino acid concentrations (42). Our results thus indicate that tRNA-dependent pre-transfer editing is non-essential, supporting our hypothesis that the synthetic site could have evolved novel features to the detriment of this quality control step. The growth of the Rosetta cells inhibited by mupirocin was not rescued by harboring the empty plasmid or the plasmid encoding EcIleRS (Fig. 5). In accordance with the previous data (36) and as shown by our kinetic analysis (Table 2), expression of ScIleRS in *E. coli* also confers mupirocin resistance to the host cells (Fig. 5*A*).

##### Rapid Hydrolysis of Val-tRNA^Ile^ in the Editing Domain

Post-transfer editing of yeast and *S. griseus* IleRS was tested independently using single-turnover deacylation assay. This approach monitors deacylation reaction at the active site and returns the rate constant that is not influenced by the product dissociation step. In this assay a limiting amount of the preformed Val-[^32^P]tRNA^Ile^ is mixed with a high surplus of the corresponding IleRS using a rapid chemical quench instrument. Our results demonstrated that both the yeast and *S. griseus* IleRS editing domains are capable of rapid hydrolysis of Val-tRNA^Ile^ (250 and 240 s^−1^, respectively; Table 4). At the level of *k*_deacyl_, they appear 5-fold more effective in valine clearance than the EcIleRS CP1 domain (24). For both enzymes, the substitution of aspartate with alanine (D334A in ScIleRS and D333A in SgIleRS) induced a 10^4^-fold drop in the rate of deacylation (Table 4). Structural analyses show that the aspartate side chain hydrogen bonds with the amino moiety of the amino acid attached to tRNA (10), providing an essential anchor of the editing substrate. The same use of the conserved aspartate is observed in ValRS (11) and LeuRS (13, 14), and it appears to be a common feature of the class Ia editing aaRS enzymes.

**TABLE 4:**
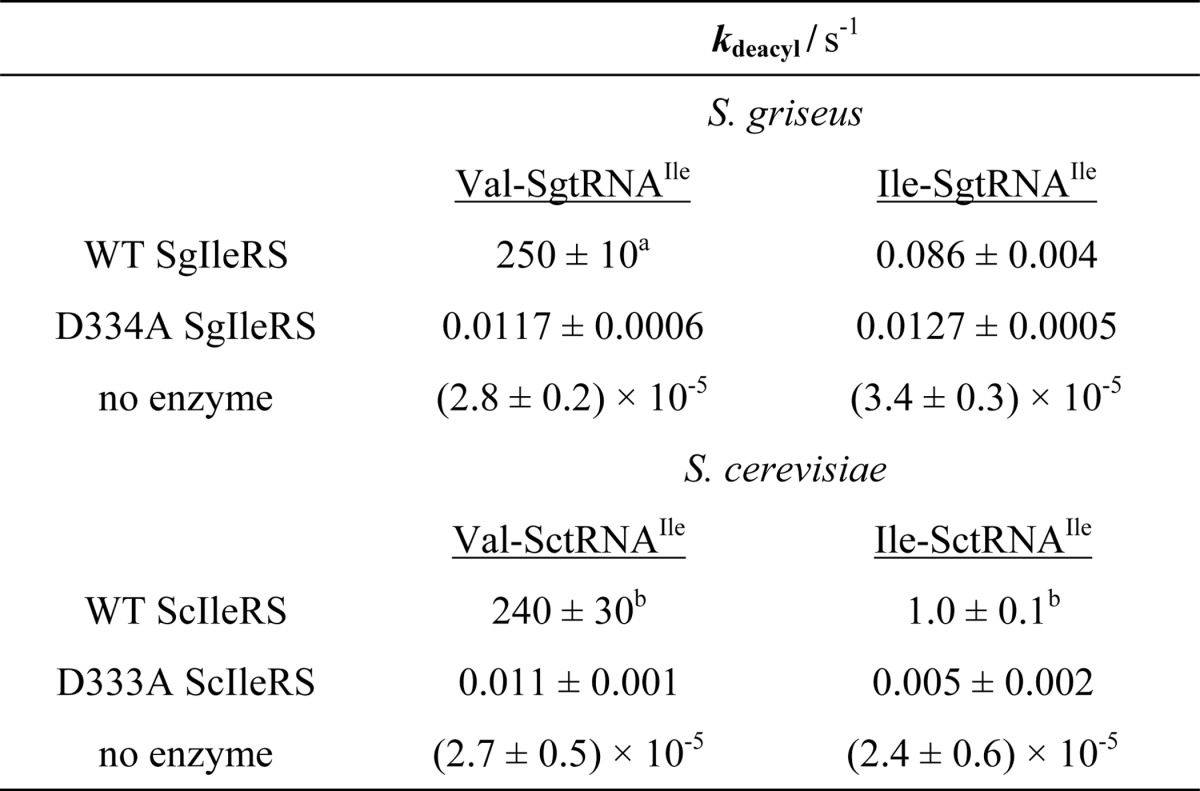
**Single-turnover deacylations by SgIleRS or ScIleRS at 30 °C** The values represent the best fit value ± S.E. of three independent experi ments. Aminoacyl-tRNAs were present at 100-500 nm and enzymes at 10-20 μm concentration.

*^a^* Data are 300 ± 20 s^−1^ at 37 °C.

*^b^* Data were determined from two independent experiments.

##### Specificity of the Editing Pathways

Our previous work (4) established that the synthetic site of EcIleRS lacks stringency in tRNA-dependent pre-transfer editing because low, but reproducible, non-stoichiometric ATP consumption was observed during cognate Ile-tRNA^Ile^ synthesis by the deacylation-defective enzyme (*k*_AMP_/*k*_Ile-tRNA_ = 1.5). To further examine characteristics of the SgIleRS synthetic site, we measured the rates of AMP formation and Ile-tRNA^Ile^ formation in parallel reactions relying on either [^32^P]ATP or [^32^P]tRNA. The deacylation-defective IleRS from *S. griseus* exhibited the same rates of AMP formation and isoleucylation (*k*_AMP_/*k*_Ile-tRNA_ = 0.98; Table 3). This confirmed that cognate isoleucine is not proofread within the synthetic site, corroborating further our conclusion that the SgIleRS·tRNA complex cannot carry out aa-AMPs hydrolysis. We further showed that the SgIleRS post-transfer editing domain displayed high specificity against cognate Ile-tRNA^Ile^ (250 s^−1^ *versus* 0.086 s^−1^; Table 4). This is in agreement with the *k*_AMP_/*k*_Ile-tRNA_ ratio of 1.05 measured in the presence of wild-type SgIleRS (Table 3).

In contrast, the yeast enzyme consumed about 1.5 molecules of ATP per Ile-tRNA^Ile^ (*k*_AMP_/*k*_Ile-tRNA_ = 1.60; Table 3), arguing for weak proofreading of isoleucine. We detected a noticeable deacylation of the cognate Ile-tRNA^Ile^ by ScIleRS under single turnover conditions (*k*_deacyl_ = 1.0 s^−1^; Table 4), which may account for the higher ATP consumption. The reason for such kinetic behavior is not yet understood. An ATP consumption in aminoacylation close to one was observed by D333A ScIleRS (*k*_AMP_/*k*_Ile-tRNA_ = 1.27; Table 3).

##### Evolutionary Origin of the Two IleRS Types

**FIGURE 6.**
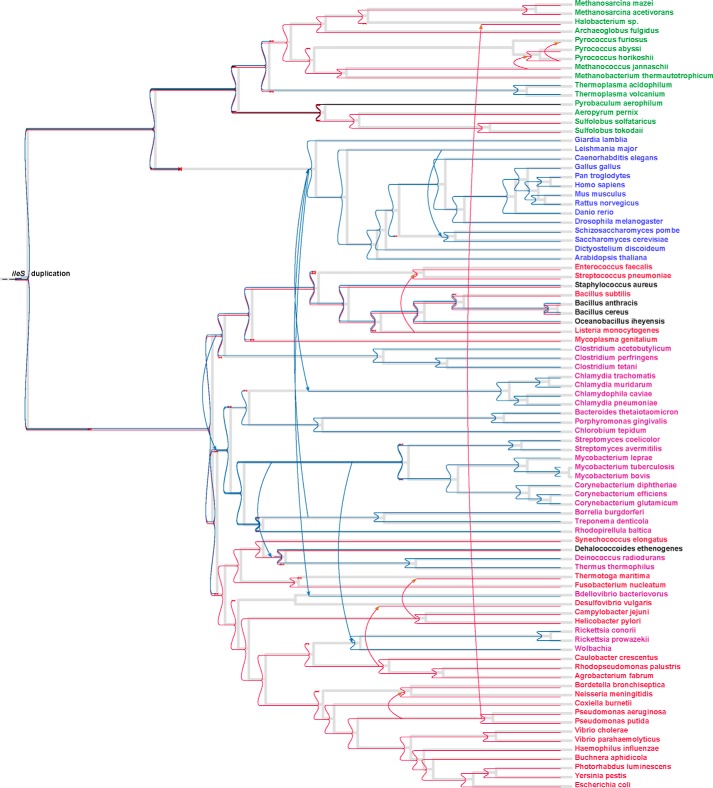
**Most parsimonious reconciliation of the IleRS gene tree and the corresponding species tree.** Species with bacterial IleRS (bIleRS1) are colored *red*; species with eukaryote-like IleRS are shown in *purple*; species with both bIleRS1 and bIleRS2 are shown in *black*; archaeal species are *green*, and eukaryotes are *blue*. The *gray lines* represent the species tree, and the *colored lines* represent evolutionary events associated with the gene tree as follows: *horizontal lines* denote speciation; *arrows* denote the most probable horizontal gene transfers that explain the occurrence of the IleRS type in a certain organism; *red cross* marks a loss of a gene; and a *blue square* (denoted also with *ileS* duplication) marks the duplication event. The *red* and *blue* colors mark the duplication event and further separation and speciation of bIleRS1 and bIleRS2, respectively.

The observed *ileS1* phylogeny is in good agreement with the previously reported analyses and with the consensus species trees (20, 35). In contrast, *ileS2* follows the species phylogeny only for separate clades at short evolutionary distances, but more distant clades show relationships that are best explained by multiple ancient horizontal transfers. The cytosolic IleRSs of all eukarya cluster as a compact monophyletic clade with the eukaryote-like enzymes, depicting their common evolutionary origin. However, it is clear from the tree that the last common ancestor of all bIleRS2 sequences pre-dates the last common ancestor of all analyzed eukarya and likely eukarya in general, suggesting that an ancestor of eukarya acquired the bIleRS2-type enzyme by horizontal transfer from a bacterial donor. This contradicts the previous proposal in which early horizontal transfer from eukarya to bacteria accounts for the occurrence of the eukaryote-like genes in the bacterial species (21, 35). Our comprehensive analysis further showed that bIleRS2 enzymes reside in a significant number of evolutionary distant clades of bacteria, including some ancient groups like clostridia (43). To get better insight into the evolutionary history of bIleRS2 and the relationship to their eukaryotic counterparts, we used an algorithm for most parsimonious reconciliations of the *ileS* gene tree and a dated species tree, using Nearest Neighbor Interchange edit operations on the edges of the gene tree with bootstrap values under 80 (bootstrap thresholds and cost parameters were varied to test the robustness of the reconciliation tree, see “Experimental Procedures”). Unexpectedly, our analysis (Fig. 6) supports a scenario in which the *ileS1* and *ileS2* genes of contemporary bacteria are the descendants of genes that might have arisen by a duplication event before the separation of bacteria and archaea. Thus, the bacterial enzyme encoded by *ileS2* appears to be of ancient bacterial origin, and it diverged from bIleRS1 through speciation. Based on the disagreement of eukaryote-like IleRS2 with the consensus bacterial phylogeny (Fig. 6) and on the fact that all eukaryote cytosolic IleRS sequences form a compact clade relatively deep inside the bacterial IleRS tree, we conclude that the horizontal gene transfer of *ileS2* from the ancient bacteria to the ancestor of eukarya more than 2.2 billion years ago is likely responsible for the acquisition of cytosolic IleRSs in contemporary eukaryotes. Also, horizontal gene transfer between bacteria appears responsible for the occurrence of *ileS2* genes in some α-proteobacteria (*e.g. Rickettsia*). It is tempting to speculate that the antibiotic resistance feature associated with the enzyme encoded by *ileS2* was an evolutionary driving force for extensive horizontal transfer of this gene. Interestingly, the Loki archaeon, the representative of the recently discovered archaeal lineage of lokiarchaeota (44), has *ileS2* in its genome in addition to an archaea-type IleRS.

## Discussion

### 

#### 

##### Synthetic and Editing Pathways Are Evolutionarily Conserved in IleRSs

IleRS is highly suitable for exploring evolution of the editing pathways in class I aaRS. Despite the existing editing domain that rapidly hydrolyzes misaminoacylated tRNA, the synthetic site of this enzyme may also noticeably contribute to editing through the tRNA-dependent pre-transfer route. This distinguishes EcIleRS from the closely related *E. coli* class Ia ValRS and LeuRS, which apparently rely exclusively on the post-transfer editing domain to ensure the accuracy of aminoacylation (3, 14, 45). Why IleRS exhibits tRNA-dependent pre-transfer editing and whether this activity is the remnant of an ancient editing or a novel feature of the synthetic site however remained elusive. Prompted by the intriguing IleRS phylogeny (Figs. 2 and 6), we sought the answer by careful kinetic analysis of the phylogenetically distinct IleRS types.

We performed a detailed steady-state and single-turnover kinetic analysis of the synthetic and editing pathways using the yeast cytosolic enzyme and eukaryote-like IleRS from the bacterium *S. griseus* as model enzymes. These enzymes appear to have a common origin (both are *ileS2*-encoded) but have diverged during evolutionary time, presumably driven in part by adaptation to their different bacterial and eukaryotic cell environments. Comparison of their contemporary features thus may provide some interesting conclusions about the interconnection of catalysis and environmental constraints. Furthermore, the differences relative to the synthetic and editing mechanisms of EcIleRS (encoded by *ileS1* gene (3, 4, 24)) shed light on the evolution of aaRS proofreading, in particular the idiosyncratic tRNA-dependent pre-transfer editing.

The inspection of sequence alignment of 334 IleRS sequences from 325 species constructed for the phylogenetic analysis reveals no relevant substitutions or deletions within the conserved regions of the post-transfer editing domain. This shows that IleRSs from all domains of life rely on the functional CP1 editing domain for the accurate decoding of isoleucine codons, as anticipated. In addition, the members of both IleRS types (EcIleRS and ScIleRS) apparently retain the capacity to use tRNA-dependent pre-transfer editing within the synthetic site. In corroboration with the presented phylogenetic analysis, this finding indicates that tRNA-dependent pre-transfer editing is an ancient feature of the IleRS synthetic site. Quite unexpectedly, this feature is lost in *S. griseus,* presumably as a consequence of the environmental pressure (see below). On the whole, our results support the notion that the synthetic pathway and origin of amino acid specificity is well conserved in IleRSs. This parallels the studies on LeuRS (46), which displays equally weak amino acid discrimination and comparable requirement for editing in the enzymes of *E. coli* and the yeast cytosol. Yet importantly, the translational quality control mechanisms may also be species-specific as reported for some class II aaRSs (47–50).

##### General Features of IleRS Editing

Similarly to some class II aaRS (51–54), IleRS appears to use post-transfer editing in *trans* to some extent (Table 3).^4^ The conclusion is supported by the unexpectedly low *k*_AMP_/*k*_Val-tRNA_ ratio and high misaminoacylation rate of SgIleRS (Table 3). ScIleRS and SgIleRS contain a comparably powerful post-transfer editing domain for preventing translational errors (Table 4). This domain acts in *cis* by hydrolyzing misaminoacylated tRNA after translocation of its 3′-end from the synthetic to the editing site (Fig. 1, *pathway 4*). Yet, if translocation occurs at a rate similar to or slower than the rate of aa-tRNA dissociation, a portion of misaminoacylated tRNA evades *cis*-editing and dissociates into solution. This fraction may rebind for editing in *trans* (Fig. 1, *pathway 5*). The low *k*_AMP_/*k*_Val-tRNA_ ratio for SgIleRS relative to ScIleRS (Table 3) is puzzling due to the presence of the efficient post-transfer editing domain in both enzymes, and it suggests that measured post-transfer editing is under-represented in this case. This is corroborated by the observation that post-transfer editing in *trans* is disfavored in the assay that measures *k*_AMP_/*k*_Val-tRNA_ ratios (14). On that ground, ScIleRS, with its tRNA-dependent pre-transfer editing, appeared as superior editing machinery than SgIleRS under conditions of double label assay. Given that pre-transfer editing clears mistakes in the synthetic site, this pathway may be valuable in cases of the slow translocation step. This may shed some light on the benefits of tRNA-dependent pre-transfer editing.

Recently we proposed that kinetic partitioning of aa-AMP between aminoacyl transfer and hydrolysis determines partitioning of the pre- and post-transfer editing pathways (3). Here, we showed that a noticeably faster transfer in yeast IleRS, relative to the bacterial counterpart, did not result in a drastic drop in pre-transfer editing. This suggests that the faster aminoacyl transfer step is accompanied by the faster aa-AMP hydrolysis, resulting in the similar level of partitioning. This is not unexpected as we demonstrated that the same amino acid residue (Tyr-59 of EcIleRS) controls both the aminoacyl transfer step and aa-AMP hydrolysis (4). Because synthetic and hydrolytic subsites are apparently highly overlapped within the IleRS synthetic site, a significant level of coupling between all opposing reactions is expected. Yet, why some enzymes in a complex with tRNA show a high propensity to pre-transfer editing, like IleRS from *E. coli* or yeast, and some not, such as EcValRS, EcLeuRS, and SgIleRS, remains obscure.

##### tRNA-dependent Pre-transfer Editing as an Optional Activity in IleRS

We showed evidence that IleRS from *S. griseus* naturally lacks tRNA-dependent pre-transfer editing. To our knowledge, this is the first report of such behavior in IleRS. Despite the lack of synthetic site editing, SgIleRS can functionally substitute endogenous IleRS in *E. coli*, under error-prone conditions where editing is essential for cell survival. This provides further evidence that tRNA-dependent pre-transfer editing is not the critical error correction step in Ile-tRNA^Ile^ synthesis. This is possible because the powerful post-transfer editing domain acts as the main cleaner of the aminoacylation errors in these enzymes. Hence, an early perspective that tRNA-dependent pre-transfer editing is the major proofreading step in IleRS (7, 15) appears to be altered toward the view that promotes an optional character of this synthetic site-based activity. However, we cannot exclude the possibility that pre-transfer editing would confer a small comparative advantage to *E. coli* that could not be ascertained in laboratory conditions.

**FIGURE 7.**
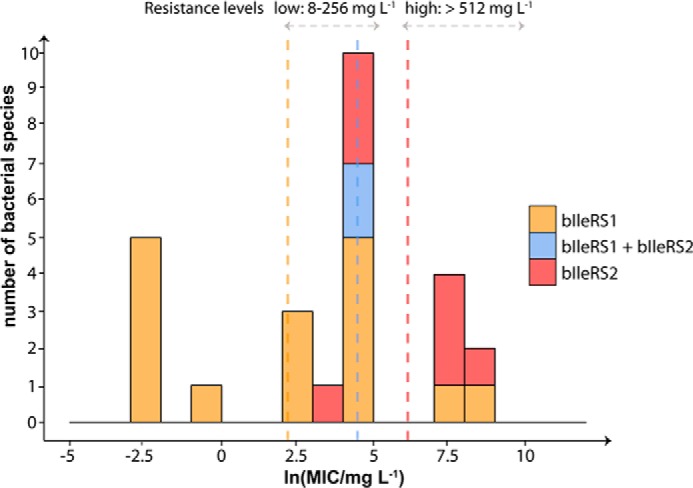
**Distribution of mupirocin MIC values for bacterial species.** The MIC values were compiled from the literature (37, 41, 47, 56), and the corresponding IleRS type was assigned according to sequence similarity and phylogenetic analysis. The range of ln(MIC) values was divided into 12 bins (bin width equals 1 unit), and the number of species in each bin was plotted on the *y* axis. The overlapping columns are shown as *stacked.* The *vertical lines* represent the mean value for each group (line colors correspond to group colors). The levels of mupirocin resistance represent the literature values for acquired resistance in *Staphylococcus* species (59).

Resistance toward mupirocin inhibition is well established for bIleRS2 enzymes (37, 41, 55). A good correlation between the minimal inhibitory concentrations (MIC) of mupirocin that inhibits the visual growth (37, 56–59) and the IleRS type present in the cell has been observed for a number of bacterial species (Fig. 7). Intriguingly the MIC values for the bIleRS2-dependent bacterial species span a considerable range in values and so do the inhibitory constants (*K_i_*) of the isolated bIleRS2 enzymes. The enzymes from *Thermus thermophilus* (TtIleRS) and *P. fluorescens* (PfIleRS2), both *ileS2*-type, exhibit the 10^4^-fold difference in the *K_i_* values (41, 60). Previous attempts to identify the synthetic site residues responsible for the antibiotic resistance did not yield a conclusive result. The structure of *S. aureus* IleRS1 (SaIleRS1) bound to tRNA^Ile^ and mupirocin (61) depicted the interactions responsible for high affinity of the synthetic site for the antibiotic. TtIleRS in a complex with mupirocin revealed a different binding pattern relative to SaIleRS1, particularly with the KMSKS motif (60). The key role in binding was initially attributed to the conserved ^585^HGF sequence (numbering according to SaIleRS1) with Phe-587 that participates in stacking interactions with the conjugated system of the nonanoic acid moiety of mupirocin. Kinetic analysis revealed that Phe-587 contribution is important but not predominant (60). Several other motifs that differ between the mupirocin-sensitive and mupirocin-resistant IleRS groups (Fig. 2*B*) were recognized, without an obvious candidate for the effect observed in SgIleRS. We noticed the motif ^554^EG (Fig. 2*B*) whose glycine hydrogen bonds to the C-6-hydroxyl group of the mupirocin pyran ring in SaIleRS1 (61). This interaction is not conserved in SgIleRS (and PfIleRS2) and thus may contribute to the established mupirocin resistance. Nevertheless, the binding determinants are likely scattered throughout the synthetic site and may even be expressed differently due to variations in the synthetic active sites.

Adaptation of the bIleRS2-type synthetic site to a better antibiotic resistance is likely driven by strong environmental pressure. Accordingly, the evolution of high resistance of SgIleRS to mupirocin (*K_i_* = 10 mm) was probably facilitated by the advantage that the antibiotic-resistant protein brings to the bacterial life in soil. In accomplishing that, the synthetic pathway was kept almost unchanged in specificity and efficiency presumably because of the strong evolutionary pressure for rapid protein biosynthesis. It is tempting to speculate that the synthetic site, on contrary, had a freedom to trade off tRNA-dependent pre-transfer editing for superior antibiotic elimination. This is consistent with the observation that dedicated post-transfer editing domain is the main gatekeeper of the accuracy of Ile-tRNA^Ile^ synthesis. The ultimate conclusion of this work is that tRNA-dependent pre-transfer editing is not an evolutionary constraint of the IleRS synthetic site.

## Author Contributions

N. C., M. D., and I. G. S. designed the study and analyzed the data. N. C. performed single-turnover kinetic analyses (Fig. 3 and Table 4), complementation assay (Fig. 5), and analyzed IleRS phylogeny (Figs. 2 and 6). M. D. performed steady-state analysis of the synthetic and editing reactions (Tables 1–3), mupirocin inhibition (Fig. 4), and complementation assay (Fig. 5). N. S. and M. B. provided technical assistance with cloning and isolation of proteins and tRNAs, and M. B. provided technical assistance with complementation. B. L. supervised N. C. in phylogenetic analysis and critically read the manuscript. I. G. S. wrote the manuscript with the contributions from all authors. All authors reviewed the results and approved the final version of the manuscript.

## Footnotes

4 N. Cvetesic and I. Gruic-Sovulj, unpublished data.

## Footnotes

3 The abbreviations used are:

aaRS: aminoacyl-tRNA synthetase
aa-AMP: aminoacyl-adenylate
aa-tRNA: aminoacyl-tRNA
bIleRS1: bacterial IleRS
bIleRS2: eukaryote-like IleRS
CP1: connective peptide 1
EcIleRS: *E. coli* IleRS
EctRNA^Ile^: *E. coli* tRNA^Ile^
Ile-tRNA^Ile^: isoleucyl-tRNA^Ile^
IleRS: isoleucyl-tRNA synthetase
IPPase: inorganic pyrophosphatase
LeuRS: leucyl-tRNA synthetase
MIC: minimal inhibitory concentration
MSA: multiple sequence alignment
Ni^2+^-NTA: nickel-nitrilotriacetic acid
PfIleRS2: *P. fluorescens* bIleRS2
PP_i_: pyrophosphate
SaIleRS1: *S. aureus* bIleRS1
ScIleRS: *S. cerevisiae* IleRS
SctRNA^Ile^: *S. cerevisiae* tRNA^Ile^
SgIleRS: *S. griseus* IleRS
SgtRNA^Ile^: *S. griseus* tRNA^Ile^
TtIleRS: *T. thermophilus* IleRS
Val-AMP: valyl-AMP
Val-tRNA^Ile^: valyl-tRNA^Ile^
ValRS: valyl-tRNA synthetase.

## References

[1] Perona J. J., and Gruic-Sovulj I. (2014) Synthetic and editing mechanisms of aminoacyl-tRNA synthetases. Top. Curr. Chem. 344, 1–41 23852030 10.1007/128_2013_456

[2] Jakubowski H. (2012) Quality control in tRNA charging. Wiley Interdiscip. Rev. RNA 3, 295–310 22095844 10.1002/wrna.122

[3] Dulic M., Cvetesic N., Perona J. J., and Gruic-Sovulj I. (2010) Partitioning of tRNA-dependent editing between pre- and post-transfer pathways in class I aminoacyl-tRNA synthetases. J. Biol. Chem. 285, 23799–23809 20498377 10.1074/jbc.M110.133553 PMC2911327

[4] Dulic M., Perona J. J., and Gruic-Sovulj I. (2014) Determinants for tRNA-dependent pretransfer editing in the synthetic site of isoleucyl-tRNA synthetase. Biochemistry 53, 6189–6198 25207837 10.1021/bi5007699 PMC4188249

[5] Nordin B. E., and Schimmel P. (2005) in Aminoacyl tRNA Synthetases (Ibba M., Francklyn C., and Cusack S., eds) pp. 24–35, Landes Biosciences, Georgetown, TX

[6] Baldwin A. N., and Berg P. (1966) Transfer ribonucleic acid-induced hydrolysis of valyladenylate bound to isoleucyl ribonucleic acid synthetase. J. Biol. Chem. 241, 839–845 5324173

[7] Fersht A. R. (1977) Editing mechanisms in protein synthesis. Rejection of valine by the isoleucyl-tRNA synthetase. Biochemistry 16, 1025–1030 321008 10.1021/bi00624a034

[8] Nureki O., Vassylyev D. G., Tateno M., Shimada A., Nakama T., Fukai S., Konno M., Hendrickson T. L., Schimmel P., and Yokoyama S. (1998) Enzyme structure with two catalytic sites for double-sieve selection of substrate. Science 280, 578–582 9554847 10.1126/science.280.5363.578

[9] Lin L., Hale S. P., and Schimmel P. (1996) Aminoacylation error correction. Nature 384, 33–34 8900273 10.1038/384033b0

[10] Fukunaga R., and Yokoyama S. (2006) Structural basis for substrate recognition by the editing domain of isoleucyl-tRNA synthetase. J. Mol. Biol. 359, 901–912 16697013 10.1016/j.jmb.2006.04.025

[11] Fukai S., Nureki O., Sekine S., Shimada A., Tao J., Vassylyev D. G., and Yokoyama S. (2000) Structural basis for double-sieve discrimination of l-valine from l-isoleucine and l-threonine by the complex of tRNA(Val) and valyl-tRNA synthetase. Cell 103, 793–803 11114335 10.1016/s0092-8674(00)00182-3

[12] Cusack S., Yaremchuk A., and Tukalo M. (2000) The 2 A crystal structure of leucyl-tRNA synthetase and its complex with a leucyl-adenylate analogue. EMBO J. 19, 2351–2361 10811626 10.1093/emboj/19.10.2351 PMC384370

[13] Lincecum T. L. Jr., Tukalo M., Yaremchuk A., Mursinna R. S., Williams A. M., Sproat B. S., Van Den Eynde W., Link A., Van Calenbergh S., Grøtli M., Martinis S. A., and Cusack S. (2003) Structural and mechanistic basis of pre- and posttransfer editing by leucyl-tRNA synthetase. Mol. Cell 11, 951–963 12718881 10.1016/s1097-2765(03)00098-4

[14] Cvetesic N., Perona J. J., and Gruic-Sovulj I. (2012) Kinetic partitioning between synthetic and editing pathways in class I aminoacyl-tRNA synthetases occurs at both pre-transfer and post-transfer hydrolytic steps. J. Biol. Chem. 287, 25381–25394 22648413 10.1074/jbc.M112.372151 PMC3408192

[15] Bishop A. C., Nomanbhoy T. K., and Schimmel P. (2002) Blocking site-to-site translocation of a misactivated amino acid by mutation of a class I tRNA synthetase. Proc. Natl. Acad. Sci. U.S.A. 99, 585–590 11782529 10.1073/pnas.012611299 PMC117349

[16] Glasfeld E., Landro J. A., and Schimmel P. (1996) C-terminal zinc-containing peptide required for RNA recognition by a class I tRNA synthetase. Biochemistry 35, 4139–4145 8672449 10.1021/bi9527810

[17] Fuller A. T., Mellows G., Woolford M., Banks G. T., Barrow K. D., and Chain E. B. (1971) Pseudomonic acid: an antibiotic produced by *Pseudomonas fluorescens*. Nature 234, 416–417 5003547 10.1038/234416a0

[18] Shiba K., Motegi H., and Schimmel P. (1997) Maintaining genetic code through adaptations of tRNA synthetases to taxonomic domains. Trends Biochem. Sci. 22, 453–457 9433122 10.1016/s0968-0004(97)01135-3

[19] Doolittle R. F., and Handy J. (1998) Evolutionary anomalies among the aminoacyl-tRNA synthetases. Curr. Opin. Genet. Dev. 8, 630–636 9914200 10.1016/s0959-437x(98)80030-0

[20] Woese C. R., Olsen G. J., Ibba M., and Söll D. (2000) Aminoacyl-tRNA synthetases, the genetic code, and the evolutionary process. Microbiol. Mol. Biol. Rev. 64, 202–236 10704480 10.1128/mmbr.64.1.202-236.2000 PMC98992

[21] Brown J. R., Zhang J., and Hodgson J. E. (1998) A bacterial antibiotic resistance gene with eukaryotic origins. Curr. Biol. 8, R365–R367 9635179 10.1016/s0960-9822(98)70238-6

[22] Yokogawa T., Kitamura Y., Nakamura D., Ohno S., and Nishikawa K. (2010) Optimization of the hybridization-based method for purification of thermostable tRNAs in the presence of tetraalkylammonium salts. Nucleic Acids Res. 38, e89 20040572 10.1093/nar/gkp1182 PMC2847242

[23] Wolfson A. D., and Uhlenbeck O. C. (2002) Modulation of tRNAAla identity by inorganic pyrophosphatase. Proc. Natl. Acad. Sci. U.S.A. 99, 5965–5970 11983895 10.1073/pnas.092152799 PMC122885

[24] Cvetesic N., Bilus M., and Gruic-Sovulj I. (2015) The tRNA A76 hydroxyl groups control partitioning of the tRNA-dependent pre- and post-transfer editing pathways in class I tRNA synthetase. J. Biol. Chem. 290, 13981–13991 25873392 10.1074/jbc.M115.648568 PMC4447971

[25] Gruic-Sovulj I., Uter N., Bullock T., and Perona J. J. (2005) tRNA-dependent aminoacyl-adenylate hydrolysis by a nonediting class I aminoacyl-tRNA synthetase. J. Biol. Chem. 280, 23978–23986 15845536 10.1074/jbc.M414260200

[26] Cvetesic N., Akmacic I., and Gruic-Sovulj I. (2013) Lack of discrimination against non-proteinogenic amino acid norvaline by elongation factor Tu from. Croat. Chem. Acta 86, 73–82 23750044 10.5562/cca2173 PMC3675784

[27] Edgar R. C. (2004) MUSCLE: multiple sequence alignment with high accuracy and high throughput. Nucleic Acids Res. 32, 1792–1797 15034147 10.1093/nar/gkh340 PMC390337

[28] Capella-Gutiérrez S., Silla-Martínez J. M., and Gabaldón T. (2009) trimAl: a tool for automated alignment trimming in large-scale phylogenetic analyses. Bioinformatics 25, 1972–1973 19505945 10.1093/bioinformatics/btp348 PMC2712344

[29] Stamatakis A. (2014) RAxML version 8: a tool for phylogenetic analysis and post-analysis of large phylogenies. Bioinformatics 30, 1312–1313 24451623 10.1093/bioinformatics/btu033 PMC3998144

[30] Nguyen T. H., Ranwez V., Pointet S., Chifolleau A. M., Doyon J. P., and Berry V. (2013) Reconciliation and local gene tree rearrangement can be of mutual profit. Algorithms Mol. Biol. 8, 12 23566548 10.1186/1748-7188-8-12 PMC3871789

[31] Ciccarelli F. D., Doerks T., von Mering C., Creevey C. J., Snel B., and Bork P. (2006) Toward automatic reconstruction of a highly resolved tree of life. Science 311, 1283–1287 16513982 10.1126/science.1123061

[32] Letunic I., and Bork P. (2007) Interactive Tree Of Life (iTOL): an online tool for phylogenetic tree display and annotation. Bioinformatics 23, 127–128 17050570 10.1093/bioinformatics/btl529

[33] Paradis E., Claude J., and Strimmer K. (2004) APE: analyses of phylogenetics and evolution in R language. Bioinformatics 20, 289–290 14734327 10.1093/bioinformatics/btg412

[34] Lundberg D. S., Lebeis S. L., Paredes S. H., Yourstone S., Gehring J., Malfatti S., Tremblay J., Engelbrektson A., Kunin V., del Rio T. G., Edgar R. C., Eickhorst T., Ley R. E., Hugenholtz P., Tringe S. G., and Dangl J. L. (2012) Defining the core *Arabidopsis thaliana* root microbiome. Nature 488, 86–90 22859206 10.1038/nature11237 PMC4074413

[35] Brown J. R., Gentry D., Becker J. A., Ingraham K., Holmes D. J., and Stanhope M. J. (2003) Horizontal transfer of drug-resistant aminoacyl-transfer-RNA synthetases of anthrax and Gram-positive pathogens. EMBO Rep. 4, 692–698 12792655 10.1038/sj.embor.embor881 PMC1326320

[36] Racher K. I., Kalmar G. B., and Borgford T. J. (1991) Expression and characterization of a recombinant yeast isoleucyl-tRNA synthetase. J. Biol. Chem. 266, 17158–17164 1910039

[37] Sassanfar M., Kranz J. E., Gallant P., Schimmel P., and Shiba K. (1996) A eubacterial *Mycobacterium tuberculosis* tRNA synthetase is eukaryote-like and resistant to a eubacterial-specific antisynthetase drug. Biochemistry 35, 9995–10003 8756461 10.1021/bi9603027

[38] Johnson K. A. (1992) in The Enzymes (Sigman D. S., ed) Vol. 20, pp. 1–61, Academic Press, Inc., San Diego

[39] Minajigi A., and Francklyn C. S. (2010) Aminoacyl transfer rate dictates choice of editing pathway in threonyl-tRNA synthetase. J. Biol. Chem. 285, 23810–23817 20504770 10.1074/jbc.M110.105320 PMC2911285

[40] Hughes J., and Mellows G. (1980) Interaction of pseudomonic acid A with *Escherichia coli* B isoleucyl-tRNA synthetase. Biochem. J. 191, 209–219 6258580 10.1042/bj1910209 PMC1162199

[41] Yanagisawa T., and Kawakami M. (2003) How does *Pseudomonas fluorescens* avoid suicide from its antibiotic pseudomonic acid? Evidence for two evolutionarily distinct isoleucyl-tRNA synthetases conferring self-defense. J. Biol. Chem. 278, 25887–25894 12672810 10.1074/jbc.M302633200

[42] Bennett B. D., Kimball E. H., Gao M., Osterhout R., Van Dien S. J., and Rabinowitz J. D. (2009) Absolute metabolite concentrations and implied enzyme active site occupancy in *Escherichia coli*. Nat. Chem. Biol. 5, 593–599 19561621 10.1038/nchembio.186 PMC2754216

[43] Jones D. T., and Keis S. (2005) in Handbook on Clostridia (Durre P., ed) pp. 3–19, Taylor & Francis Group, Boca Raton, FL

[44] Spang A., Saw J. H., Jørgensen S. L., Zaremba-Niedzwiedzka K., Martijn J., Lind A. E., van Eijk R., Schleper C., Guy L., and Ettema T. J. (2015) Complex archaea that bridge the gap between prokaryotes and eukaryotes. Nature 521, 173–179 25945739 10.1038/nature14447 PMC4444528

[45] Cvetesic N., Palencia A., Halasz I., Cusack S., and Gruic-Sovulj I. (2014) The physiological target for LeuRS translational quality control is norvaline. EMBO J. 33, 1639–1653 24935946 10.15252/embj.201488199 PMC4194098

[46] Yao P., Zhou X. L., He R., Xue M. Q., Zheng Y. G., Wang Y. F., and Wang E. D. (2008) Unique residues crucial for optimal editing in yeast cytoplasmic leucyl-tRNA synthetase are revealed by using a novel knockout yeast strain. J. Biol. Chem. 283, 22591–22600 18550527 10.1074/jbc.M801181200

[47] Reynolds N. M., Ling J., Roy H., Banerjee R., Repasky S. E., Hamel P., and Ibba M. (2010) Cell-specific differences in the requirements for translation quality control. Proc. Natl. Acad. Sci. U.S.A. 107, 4063–4068 20160120 10.1073/pnas.0909640107 PMC2840081

[48] SternJohn J., Hati S., Siliciano P. G., and Musier-Forsyth K. (2007) Restoring species-specific posttransfer editing activity to a synthetase with a defunct editing domain. Proc. Natl. Acad. Sci. U.S.A. 104, 2127–2132 17283340 10.1073/pnas.0611110104 PMC1892958

[49] Ahel I., Korencic D., Ibba M., and Söll D. (2003) Trans-editing of mischarged tRNAs. Proc. Natl. Acad. Sci. U.S.A. 100, 15422–15427 14663147 10.1073/pnas.2136934100 PMC307583

[50] Hussain T., Kruparani S. P., Pal B., Dock-Bregeon A. C., Dwivedi S., Shekar M. R., Sureshbabu K., and Sankaranarayanan R. (2006) Post-transfer editing mechanism of a d-aminoacyl-tRNA deacylase-like domain in threonyl-tRNA synthetase from archaea. EMBO J. 25, 4152–4162 16902403 10.1038/sj.emboj.7601278 PMC1560354

[51] Ling J., So B. R., Yadavalli S. S., Roy H., Shoji S., Fredrick K., Musier-Forsyth K., and Ibba M. (2009) Resampling and editing of mischarged tRNA prior to translation elongation. Mol. Cell 33, 654–660 19285947 10.1016/j.molcel.2009.01.031 PMC2944653

[52] Vargas-Rodriguez O., and Musier-Forsyth K. (2013) Exclusive use of trans-editing domains prevents proline mistranslation. J. Biol. Chem. 288, 14391–14399 23564458 10.1074/jbc.M113.467795 PMC3656294

[53] An S., and Musier-Forsyth K. (2005) Cys-tRNA(Pro) editing by *Haemophilus influenzae* YbaK via a novel synthetase. YbaK.tRNA ternary complex. J. Biol. Chem. 280, 34465–34472 16087664 10.1074/jbc.M507550200

[54] Novoa E. M., Vargas-Rodriguez O., Lange S., Goto Y., Suga H., Musier-Forsyth K., and Ribas de Pouplana L. (2015) Ancestral AlaX editing enzymes for control of genetic code fidelity are not tRNA-specific. J. Biol. Chem. 290, 10495–10503 25724653 10.1074/jbc.M115.640060 PMC4400357

[55] Thomas C. M., Hothersall J., Willis C. L., and Simpson T. J. (2010) Resistance to and synthesis of the antibiotic mupirocin. Nat. Rev. Microbiol. 8, 281–289 20190824 10.1038/nrmicro2278

[56] Sutherland R., Boon R. J., Griffin K. E., Masters P. J., Slocombe B., and White A. R. (1985) Antibacterial activity of mupirocin (pseudomonic acid), a new antibiotic for topical use. Antimicrob. Agents Chemother. 27, 495–498 3923922 10.1128/aac.27.4.495 PMC180082

[57] Paulander W., Maisnier-Patin S., and Andersson D. I. (2007) Multiple mechanisms to ameliorate the fitness burden of mupirocin resistance in *Salmonella typhimurium*. Mol. Microbiol. 64, 1038–1048 17501926 10.1111/j.1365-2958.2007.05713.x

[58] Serafini F., Bottacini F., Viappiani A., Baruffini E., Turroni F., Foroni E., Lodi T., van Sinderen D., and Ventura M. (2011) Insights into physiological and genetic mupirocin susceptibility in bifidobacteria. Appl. Environ. Microbiol. 77, 3141–3146 21421794 10.1128/AEM.02540-10 PMC3126397

[59] Hetem D. J., and Bonten M. J. (2013) Clinical relevance of mupirocin resistance in *Staphylococcus aureus*. J. Hosp. Infect. 85, 249–256 24144552 10.1016/j.jhin.2013.09.006

[60] Nakama T., Nureki O., and Yokoyama S. (2001) Structural basis for the recognition of isoleucyl-adenylate and an antibiotic, mupirocin, by isoleucyl-tRNA synthetase. J. Biol. Chem. 276, 47387–47393 11584022 10.1074/jbc.M109089200

[61] Silvian L. F., Wang J., and Steitz T. A. (1999) Insights into editing from an ile-tRNA synthetase structure with tRNAIle and mupirocin. Science 285, 1074–1077 10446055

